# The Photometric Testing of High-Resolution Digital Cameras from Smartphones—A Pilot Study

**DOI:** 10.3390/s24216936

**Published:** 2024-10-29

**Authors:** Sławomir Zalewski, Krzysztof Skarżyński

**Affiliations:** Lighting Technology Division, Electrical Power Engineering Institute, Warsaw University of Technology, Koszykowa 75, 00-662 Warsaw, Poland; slawomir.zalewski@pw.edu.pl

**Keywords:** photometry, lighting measurement, smartphone, digital camera, matrix sensor, image sensor, light response, linearity response, spectral mismatch, luminance

## Abstract

Luminance is the fundamental photometric quantity representing the technical meaning of brightness. It is usually measured from a distance using a matrix sensor, which is the basis of the professional instrument. However, specific technical requirements must be fulfilled to achieve accurate results. This paper considers whether modern high-resolution smartphone cameras are suitable for luminance measurements. Three cameras from high-end smartphones were evaluated on a dedicated laboratory stand. The sensors’ output characteristics showed relatively good linearity of the individual R, G, and B channels. Unfortunately, the spectral sensitivities were unfavorable, as the minimum error achieved was about 17%. This device is classified outside the generally accepted quality scale for photometric instruments. The presented investigation confirmed that none of the high-resolution smartphone cameras tested was suitable for use as a universal luminance camera. However, one of the test devices can be developmental if restrictively calibrated and used only in a specialistic laboratory stand. Using a smartphone (or only its camera) for luminance measurements requires proper advanced calibration. It is possible, but it limits us to only dedicated applications. The pilot study presented in this paper will help create a suitable test stand for spectacle vision systems, e.g., virtual reality equipment.

## 1. Introduction

### 1.1. Smartphones in Visible Radiation Measurements

Recently, smartphones have been used increasingly often to measure various physical quantities and technical parameters. Measurements made with them appear even in formal scientific research [1,2,3,4]. There are many reasons to use smartphones as measurement devices, including:

- their common occurrence and market availability,
- the increasing number and quality of sensors and recorders in smartphones,
- the ease of collecting and transmitting data with smartphones,
- rapidly improving technical parameters of components used to manufacture smartphones,
- much lower price compared to professional research instruments.

Over time, the camera has become a permanent feature in smartphones. The size of the camera module built into the smartphone and the aperture of its optical system predispose it to specific measurement applications. It is used primarily where the low weight and size of the measuring device are critical, and where the difference between the aperture of the optical system of the meter and the diameter of the pupil of the human eye will have a significant impact on the discrepancy between the measurement and the impression of brightness felt by the observer. The difference between the aperture of the meter’s optical system and the diameter of the human eye also significantly impacts the discrepancy between the measurement and the impression of brightness felt by the observer. It determines the usability of miniature digital cameras from smartphones, such as measurement and research. 

For the reasons mentioned above, free and paid programs offering measurement of various parameters, including visible radiation, are appearing on the mobile phone application market [5,6]. The largest category of such applications measure illuminance. There are also applications offering luminance distribution measurement using cameras built into mobile phones and applications for measuring light color: tristimulus values and correlated color temperature. However, the operation of these applications mainly depends on the quality of image processing by the camera’s optical system, photosensitive matrix, and image processor [7,8,9]. Differences in data processing by individual devices will result in different values indicated by them, obtaining fundamentally different values of the measured photometric quantity, and leading to significant measurement error and a lack of credibility. Nevertheless, it can be observed that smartphones are used for lighting measurements, as it seems to be a cheap alternative to professional instrumentation, which is very expensive. 

### 1.2. Luminance Measurement: Challenges and Requirements

Luminance is the photometric quantity that describes the density of luminous intensity with respect to the projected area in a specified direction at a specified point on a real or imaginary surface (1) [10].

(1) $$L\left(C,\gamma \right)=\frac{dI\left(C,\gamma \right)}{dS\prime }=\frac{dI\left(C,\gamma \right)}{dScos\varepsilon }\left[\frac{cd}{m^{2}}\right]$$

*where:*
*L(C,γ)—is the luminance from a specified direction [cd/m^2^],*
*I(C,γ)—is the luminous intensity at the specified direction [cd],*
*(C,γ)—is the specified direction [-],*
*dS’—is the imaginary area [m^2^],*
*dS—is the real area [m^2^],*
*ε—is the angle between the normal to the area at the specified point and direction [-].*

It should be emphasized that luminance is a technical parameter closely related to the observer’s brightness perception. It also depends on the eye’s adaptation state and contrast in the field of view [11,12,13,14]. There are normative requirements for luminance in lighting systems, for example, in the case of road lighting and floodlighting [15,16]. The luminance level also affects issues related to visual disturbance, i.e., glare, which can create safety issues at work, for road traffic, or emergency lighting [17,18,19]. In recent years, scientists have focused mainly on developing appropriate measurement methodologies and investigating the primary sources of errors in luminance measurements [20,21]. These errors arise primarily in relation to the measurement of sources with a large luminance gradient [22,23], luminance modeling of optical systems of lighting fixtures [24], analysis of various lighting installations using luminance meters [25,26,27], and even luminance analysis of the night sky and urban space in terms of light pollution [28,29,30,31].

Currently, digital meters are used for luminance measurements. They can be divided into two groups: so-called “point” luminance meters with a single defined field of view, and matrix luminance meters, also known as luminance cameras. Some of these meters are built based on appropriately calibrated digital cameras [32], but it is worth noting that such solutions are characterized by very high measurement inaccuracy, reaching even 13%, depending on the spectral distribution power distribution of light [33].

**Figure 1:**
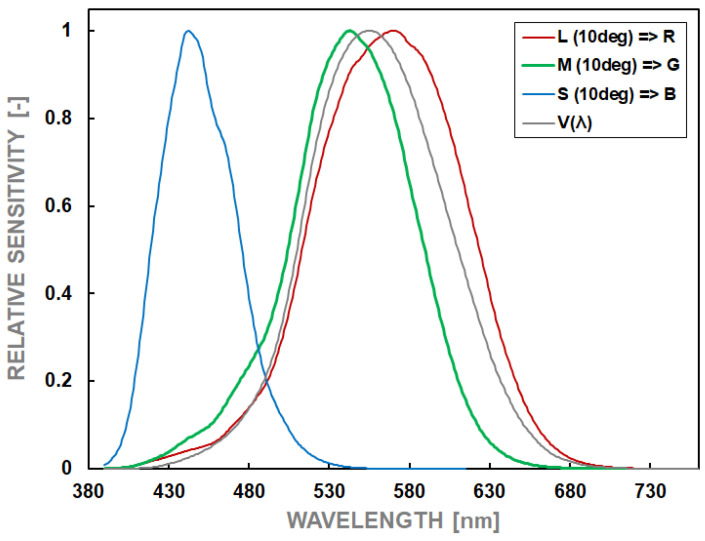
Relative spectral sensitivities of L, M, and S receptors and photopic vision of the human eye.

Accordingly, if a smartphone camera is to be used reliably as a matrix luminance meter, it should meet several essential factors. These have been described in detail in publications of the International Commission on Illumination (CIE), e.g., “Characterization of the performance of illuminance meters and luminance meters” [34]. Those factors can be divided into critical factors and secondary factors. The first ones must be fulfilled unconditionally, whereas the fulfillment of secondary factors only increases the meter’s functionality and facilitates its programming and use. These factors will be briefly described below. The first and most crucial factor is excellent spectral matching, i.e., the spectral sensitivity of individual pixels consistent with the spectral sensitivity of the human eye’s L, M, and S receptors [35]. Thanks to this, it is possible to reproduce the photopic spectral efficiency of the human eye (so-called V_λ_ (λ) curve) with high accuracy. It fundamentally influences the correlation of the measured luminance value with the human’s perception of brightness. The characteristics of spectral sensitivity for a standardized 10-degree photometric observer and the photopic spectral sensitivity of the human eye are presented in Figure 1.

Measurement inaccuracy resulting from imperfect spectral matching of the meter is defined by the spectral mismatch error f_1_′ [34]. It is described by Formula (2). Suppose the meter does not sufficiently match the V(λ). In that case, any deviation of the spectral power distribution of light emitted by the tested surface from the spectral power distribution of light with which the meter was calibrated causes significant measurement errors.

(2) $$f_{1}^{\prime }=\frac{\int_{380}^{760}\left|V_{D}\left(\lambda \right)-V_{\lambda }\left(\lambda \right)\right|d\lambda }{\int_{380}^{760}V_{\lambda }\left(\lambda \right)d\lambda }\;100\left[\%\right]$$

*where:*

$f_{1}^{\prime }$ *—is the spectral mismatch error [%],*

$V_{D}\left(\lambda \right)$ *—is the spectral sensitivity of the tested photometer’s head [-],*

$V_{\lambda }\left(\lambda \right)$ *—is the photopic spectral efficiency for the human eye [-],*

λ *—is the current wavelength in the visible range from 380 nm to 760 nm.*

The linearity response is the second critical factor for using a camera as a luminance meter, and in general it is crucial for any light measurement sensors [36,37]. It can be related to the measured luminance value or knowledge of the characteristics of this dependence. The measurement error resulting from the nonlinearity of the meter is designated as f_3_.

In addition, the angular resolution of the camera should allow for the reproduction of an image in a quality corresponding to the maximum resolution of the human eye, which is about 0.45 arc minutes [11]. Therefore, wide-angle cameras with high or very high resolution as luminance meters are advisable.

A vital camera parameter that does not significantly affect accuracy or ease of data processing is the uniformity of the matrix sensitivity. This parameter is divided into two components: vignetting effect and the individual deviation of a given pixel’s sensitivity from the average pixel sensitivity in a given area of the matrix. The vignetting effect, which is the result of the finite dimensions of the actual components of the optical system, can be measured, described numerically, and corrected with a coefficient dependent on the distance of a given pixel from the optical axis of the measuring system. On the other hand, the individual deviation of the response of a given pixel from the average is noise, and is practically impossible to correct. The only practical way to eliminate this error is to establish the minimum number of pixels from which the average value is calculated, representing the response of the matrix in a given area.

To reduce measurement inaccuracy, the above-described factors, and the two most important errors, f_1_′ and f_3_, the smartphone sensor should also meet other requirements for photometric heads. The most critical errors affecting photometers resulting from various aspects of use include [33]:

- The error resulting from the response to ultraviolet radiation is marked with the f_UV_, while the error resulting from the head’s response to infrared radiation is marked with the f_IR_. It means that the smartphone digital camera should be completely insensitive to electromagnetic radiation outside the visible range.
- The spatial correction error f_2_ indicates the radiation collection from the entire hemisphere. According to the changeable field of view, this error does not apply to luminance meter purposes.
- The error related to the accuracy of reading values from the display or analog scale is marked with the f_4_. In this case, this error results from the number of bits used to record the recorded value and the rounding of the values provided.
- The error related to changes in the meter reading over time despite measuring a constant value, i.e., repeatability of the measured value, is called fatigue error and is marked with the f_5_.
- The errors related to the effect of ambient temperature and humidity changes on the reading are marked with the f_6T_ and f_6H_, respectively.
- The error resulting from the variability of the signal measured over time (flickering) is called modulation error, and is marked with the f_7_.
- The meter error related to the polarization of the measured radiation is marked with the f_8_.
- The error related to changes in the meter range is marked with the f_11_. In the case of a digital camera, this error describes the mistake resulting from changes in the exposure time and the aperture value.
- The error related to changes in the distance from which the luminance is measured is called viewing distance error, and is marked with f_12_.
- The error associated with meter calibration is called matching error and is marked with the f_adj_.

Another condition that must be met to try to use a given camera as a luminance meter is that the electronics and software allow access to the exposure value of individual pixels read directly from the matrix without interference from the image processor. Therefore, the files cannot be processed and subjected to lossy compression. Thus, files in the *.jpg format are not suitable for processing, and access to RAW files is necessary for the universal digital negative format *.dng.

### 1.3. The Main Research Goal

As previously demonstrated, the problems of ensuring appropriate conditions for photometric measurements, including the requirements for photometer heads and luminance meters, are very complex. Thus, the main goal of this paper is to check whether using a smartphone for luminance measurements and, as a result, analyzing the light environment makes sense. The research presented here can be classified as a pilot study because digital cameras from selected phones were checked for two critical measurement factors: linearity response of the sensors’ channels and spectral mismatch. An important novelty of this research is to check the operation of modern digital sensors with very high resolution; the authors believe this is the first scientific paper to do so. Additionally, it should be emphasized that this type of matrix sensor could be suitable for use in measuring lighting parameters from drones or satellites due to their miniature construction.

## 2. Materials and Methods

### 2.1. Samples

Research on the possibilities of using a smartphone camera as a luminance meter has been limited to a few premium, but also freely available on the market devices (Table 1). Those smartphones must meet the requirement of the availability of graphic files in the appropriate format, not compressed files (RAW).

The first smartphone is a slightly outdated device from the business line of a leading manufacturer. In the research, it was marked as “Smartphone 1”. It has a camera of the manufacturer’s design with a matrix resolution of 12.1 Mpx (4024 × 3016) and 10-bit dynamics. In addition to the standard *.jpg format, this smartphone offers the generation of graphic files in the *.dng format at the user’s request.

The second smartphone, marked as “Smartphone 2”, is the successor of Smartphone 1 at the time of purchase in February 2024. It is the latest model of this manufacturer. It has a camera of the manufacturer’s design with a matrix resolution of 200 Mpx. After purchasing the phone, it turned out that the declared resolution is only available in graphic files in *.jpg format. Photos saved in *.dng format have a resolution of 4000 × 3000 pixels, i.e., 12 Mpx, and only 8-bit dynamics. A serious disadvantage of this camera is that photos in *.dng format are converted to RGB, which means a process reverse to the Bayer pattern. Moreover, this device does not provide the ability to read raw data directly from the matrix. Despite these discriminating disadvantages, the test results of the camera installed in this smartphone are presented later in the article to show the measurement discrepancies.

The third and last of the smartphones tested, marked in the study as “Smartphone 3”, is the flagship model of a manufacturer aspiring to be the leading brand. This smartphone has a camera, and a CMOS matrix manufactured by OmniVision (Santa Clara, CA, USA). The photosensitive matrix in the advertising brochures is described as Light Fusion 900 and works with the Summilux optics designed by Leica (Sydney, NSW, Australia). A comparison of the photo parameters with the catalog data indicates that it is an OV50H matrix with a native resolution of 50 Mpx. *.dng files are saved in the phone’s memory with a maximum resolution of 4088 × 3064 pixels, corresponding to a resolution of 12.5 Mpx. The sensor dynamics provided by the phone manufacturer is 12-bit. According to the matrix manufacturer’s data, it can save data with 14-bit dynamics. Comparing the parameters obtained from the smartphone with the catalog data available on the matrix manufacturer’s website shows that the capabilities of this matrix have been software-limited by the smartphone manufacturer. It is both in terms of resolution and dynamics, and it is most likely to save space in the smartphone’s memory.

### 2.2. Laboratory Setup

**Figure 2:**
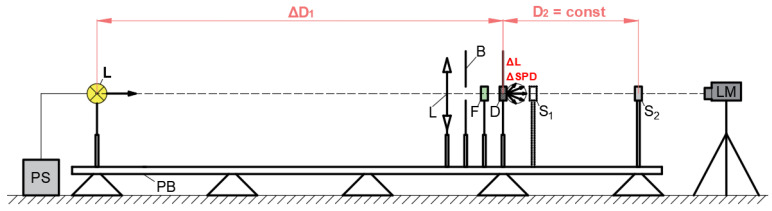
The scheme of the laboratory stand: PB—photometric bench, PS—power supply, L—light source (illuminant A—2856K), L—focusing lens, B—baffle, F—interference filter, D—diffuser, S_1_—place for spectroradiometer head, S_2_—place for the smartphone, LM—luminance meter, ΔD_1_—changeable distance between light source and diffuser to provide proper luminance value, D_2_—constant distance between diffuser and smartphone, ΔL—changeable luminance (for linearity), ΔSPD (for spectral sensitivity).

The tests of selected cameras were limited to two factors critically responsible for the quality of the data obtained and fundamentally influencing the possibility of those cameras being used for measurement purposes. The following tests were conducted: the linearity response of the central part of the matrix due to the change in the luminance level and spectral sensitivity of the selected group of pixels located in the central area of the matrix. For this purpose, a measurement stand was prepared and used in a photometric darkroom, which is schematically displayed in Figure 2.

The linearity characteristics of the matrices were determined based on a series of photographs taken independently with each camera. The photos were obtained by processing them using smartphone hardware and software. The same areas in subsequent photos were analyzed to avoid image enhancement, vignetting effect, etc. A surface with variable luminance and a constant correlated color temperature of 2856 K was photographed (marked in Figure 2 as D). The photographed surface was a two-layer transmission diffuser illuminated from behind by an incandescent light source from a variable distance. The luminance value obtained, and control of the correlated color temperature constancy, were determined using a Konica-Minolta CS-200 point luminance meter with a tristimulus values measurement function. The measurement accuracy declared by the manufacturer is ±2% ±1 digit [38]. Measurements were started at the luminance level, causing matrix saturation in the area used for measurement. In subsequent steps, the luminance was reduced to approximately 0.8 of the previous exposure value.

To easily compare individual cameras, all had the same exposure parameters set: ISO 100 matrix sensitivity and 1/20 s exposure time. A long exposure time was chosen to eliminate the potential impact of light flickering on the measurement. Unfortunately, individual cameras had different apertures. The camera in Smartphone 1 has a variable aperture with an adjustment range of f/1.5–f/2.4. For the duration of the measurements, the aperture was set to a value equal to f/1.5. The aperture in the camera mounted in Smartphone 2 is a non-adjustable parameter and has a value of f/1.7. The camera in Smartphone 3 has a fixed aperture of f/1.6. In all measurements, the zoom was set to the smallest focal length, offering the most expansive field of view of the camera. To eliminate errors resulting from different pixel sensitivities in other areas of the matrix, the cameras were immobilized for the duration of the measurements, and the data were read from pixels with the same positions in subsequent photos. The RawTherapee 5.9 free software was used to analyze the images. This area overlapped with the measurement area of the luminance meter used for the 1⁰ field of view. The meter used as a reference in the measurements was set so that the angle between the directions of observation of the surface measured by the meter and the smartphone’s camera was constant and as small as possible.

**Figure 3:**
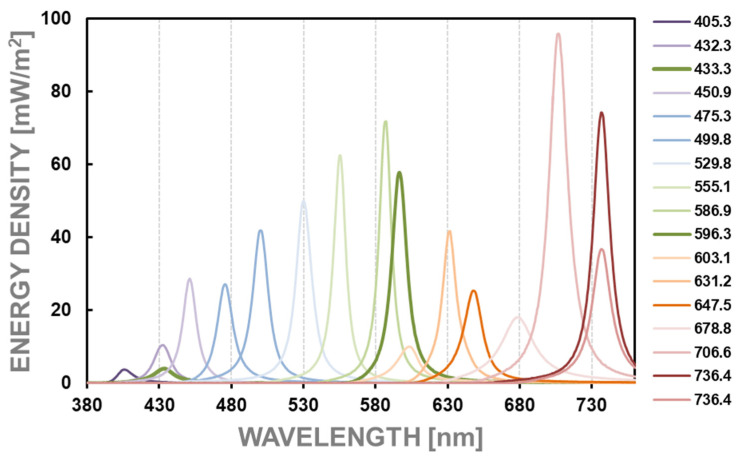
The spectral power densities emitted by the test surface using different interference filters.

The spectral sensitivity characteristics of selected pixels of the tested devices were performed using quasi-monochromatic light obtained using a set of 15 interference filters and a tungsten lamp (illuminant A). Nominally, the interference filters transmitted spectral lines from the visible range approximately every 25 nm from 390 nm to 740 nm. The spectral power densities emitted by the test surface using different interference filters are presented in Figure 3. The measurement system consisted of a light source in the form of a halogen bulb, a scattering surface, a focusing lens, interference filters, a spectroradiometer, and many diaphragms eliminating scattered light. The Spectis 4.0 spectroradiometer from GL Optic with a head collecting radiation in a directional manner and a measurement accuracy of ±3% in the visible range was used as a reference meter [39].

## 3. Results and Discussion

**Figure 4:**
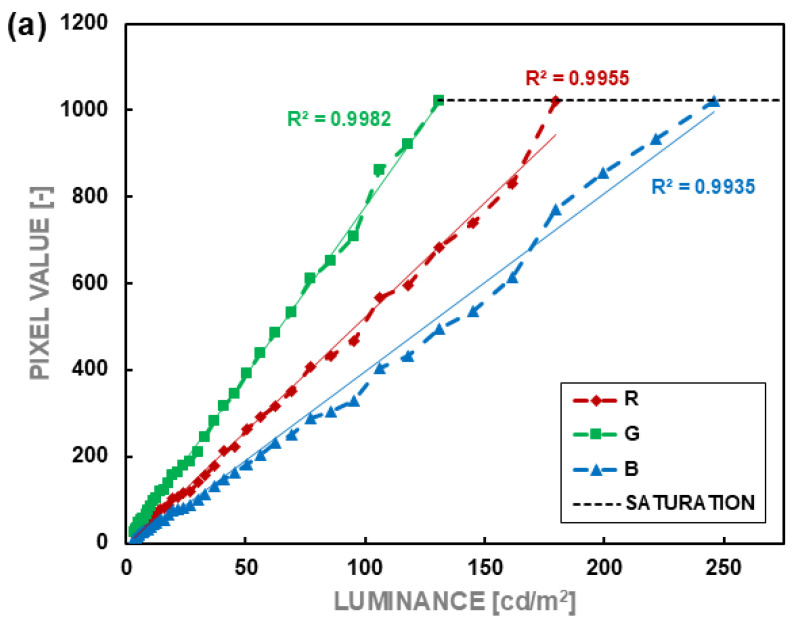

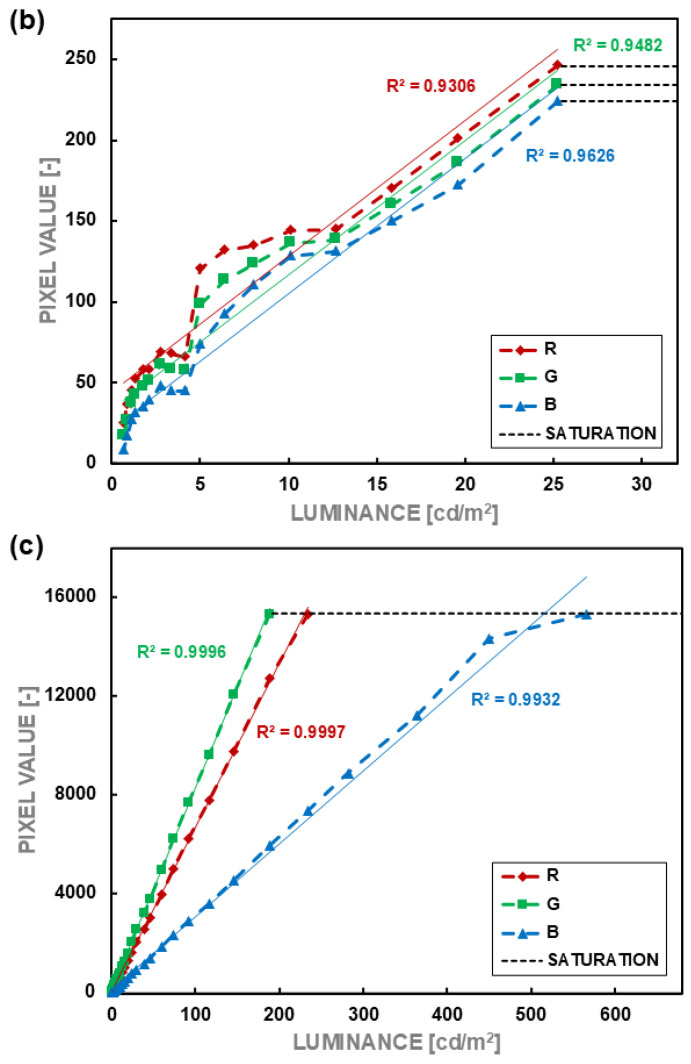
Linearity responses for channels of tested smartphones: (**a**) Smartphone 1, (**b**) Smartphone 2, (**c**) Smartphone 3.

**Figure 5:**
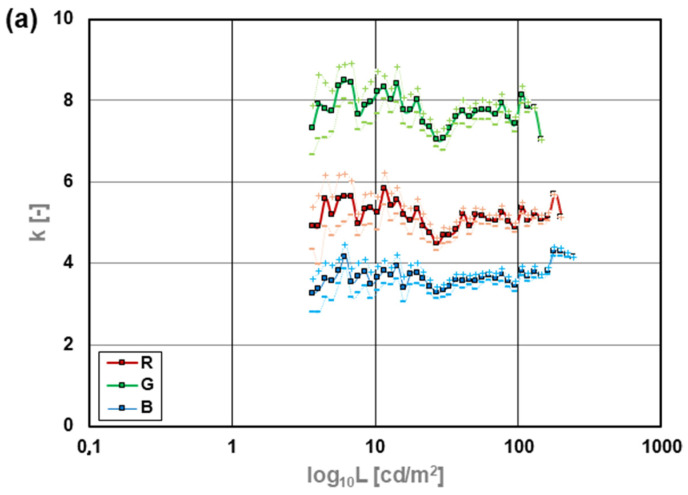

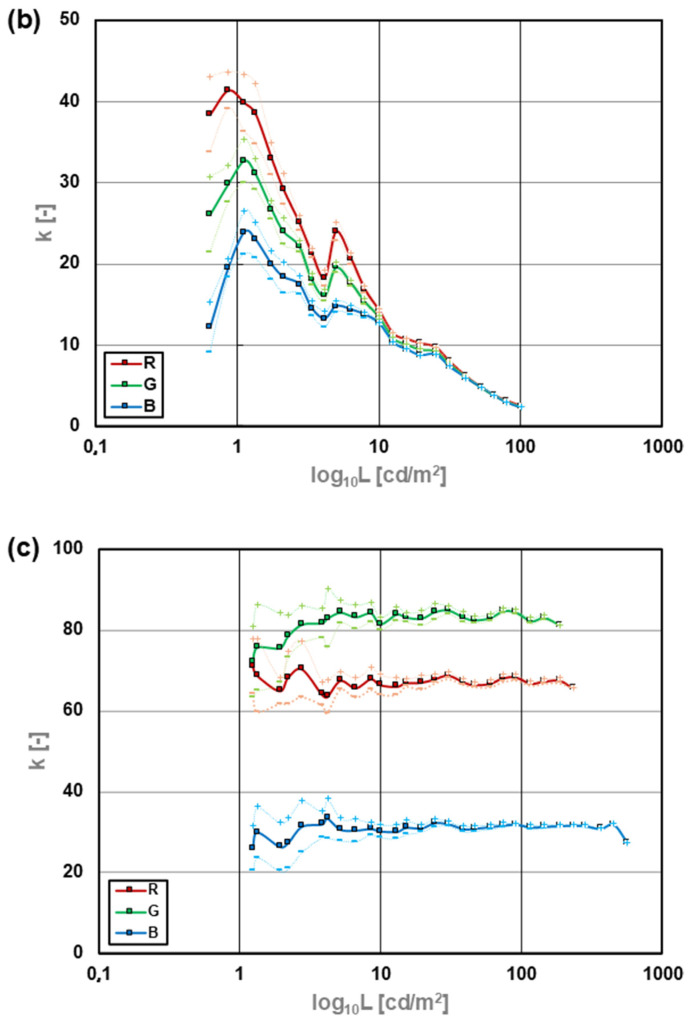
The value of the proportionality k-factor as a function of the logarithm of luminance for the smartphones tested: (**a**) Smartphone 1, (**b**) Smartphone 2, (**c**) Smartphone 3.

Based on the photographs of surfaces with different luminance values, average values of each of the color components R, G, and B of the matrix response were plotted as a function of the luminance of the photographed surface. The averaging area included 12 pixels with R, G, and B sensitivity characteristics. The selected pixels constituted a compact fragment of the matrix. In addition to the average response value, standard deviations were calculated for each group. The data were visualized in graphs. Figure 4 shows the dependencies of the matrix response on changes in the photographed luminance. The characteristics of the proportionality conversion factor k were also plotted as a function of the logarithm of luminance (3), presented in Figure 5.

(3) $$k=\frac{N}{L}\left[\frac{bit}{cd\;m^{-2}}\right]$$

(4) $$k\pm \Delta k=\frac{N\pm \Delta N}{L}\left[-\right]$$

*where:*
*L—is the luminance value of photographed surface,*
*k—is the proportionality conversion factor,*
*Δk—is the deviation of the proportionality conversion factor k,*
*N—is the pixel value from the matrix,*
*ΔN—is the standard deviation of the individual pixel responses in the tested population.*

The camera from Smartphone 1 shows quite a good linearity (Figure 4a) of the matrix response to luminance change. In this case, the most extensive linearity range was obtained for channel B (up to about 250 cd/m^2^). In comparison, the smallest was only 130 cd/m^2^ for channel G. The channels were saturated above the indicated luminance levels. The k-factor of the matrix response to luminance (Figure 5a) shows a standard deviation of 7% of the average value for the R component, 13% for the G component, and 7% for the B component.

In the case of Smartphone 2, the linearity of the matrix response is much worse. For each channel, the linearity range is only from 0.1 cd/m^2^ to only 25 cd/m^2^ (Figure 4b). On the one hand, this range would meet the requirements for outdoor lighting installations, where several to a dozen or so cd/m^2^ are expected. On the other hand, considering the luminance gradients that can occur, especially in the case of LED lighting equipment, the measurement range is too small for this smartphone to be universally useful as a luminance meter. In this case, the narrow range is due to the very high processing of the direct signal from the matrix. The information saved in *.dng files is too highly processed and, as a result, very distorted in terms of use for specialist photometric measurements.

The camera in Smartphone 3 is also characterized by quite good linearity of the response of individual channels (Figure 4c). Like the camera in Smartphone 1, channel G ranges from about 0.1 cd/m^2^ to about 200 cd/m^2^. However, the range of channel B is more extensive and reaches about 550 cd/m^2^. In contrast to Smartphone 1, channel B’s saturation occurred at twice the luminance level. Unfortunately, other channels became saturated at around 200 cd/m^2^. It could negatively impact this device’s general performance for lighting measurements. Smartphone 3 provided data with a standard deviation of 3% of the average value for channel R, 5% for channel G, and 6% for channel B (Figure 5c). The obtained data give hope for obtaining reliable information about the measured luminance.

The spectral sensitivity characteristics of individual pixels were determined based on high-luminance surface images taken through seventeen interference filters, whose spectral power densities are presented in Figure 3. It was also done using the measurement stand shown in Figure 2. Similarly, to determine matrix linearity, the analysis was conducted on the area of 36 pixels, 12 of R, G, and B characteristics, respectively.

As mentioned earlier, the spectral mismatch error of a single-channel photometer’s head, marked with the symbol f_1_′, is determined from the relationship (2). In the case of using a three-channel system with three different spectral characteristics corresponding to three types of color receptors in the human eye, it is necessary to assess the compliance of the spectral sensitivity of each channel with the appropriate kinds of receptors. To evaluate the compliance of the spectral sensitivity of individual pixels with the spectral sensitivity of receptors in the human eye, Formula (2) was modified to the relationships (5), (6), and (7).

(5) $$f_{1R}^{\prime }=\frac{\int_{380}^{760}\left|V_{R}\left(\lambda \right)-V_{L}\left(\lambda \right)\right|d\lambda }{\int_{380}^{760}V_{L}\left(\lambda \right)d\lambda }\;100\left[\%\right]$$

(6) $$f_{1G}^{\prime }=\frac{\int_{380}^{760}\left|V_{G}\left(\lambda \right)-V_{M}\left(\lambda \right)\right|d\lambda }{\int_{380}^{760}V_{M}\left(\lambda \right)d\lambda }\;100\left[\%\right]$$

(7) $$f_{1B}^{\prime }=\frac{\int_{380}^{760}\left|V_{B}\left(\lambda \right)-V_{S}\left(\lambda \right)\right|d\lambda }{\int_{380}^{760}V_{S}\left(\lambda \right)d\lambda }\;100\left[\%\right]$$

*where:*

$f_{1R}^{\prime }$ *—is the spectra mismatch error for R channel [%],*

$V_{R}\left(\lambda \right)$ *—* *is the spectral sensitivity of R-type pixel [-],*

$V_{L}\left(\lambda \right)$ *—is the spectral sensitivity of L receptors [-],*

$f_{1G}^{\prime }$ *—is the spectra mismatch error for G channel [%],*

$V_{G}\left(\lambda \right)$ *—* *is the spectral sensitivity of G-type pixel [-],*

$V_{M}\left(\lambda \right)$ *—is the spectral sensitivity of M receptors [-]*

$f_{1B}^{\prime }$ *—* *is the spectra mismatch error for B channel [%],*

$V_{B}\left(\lambda \right)$ *—is the spectral sensitivity of B-type pixel [-],*

$V_{S}\left(\lambda \right)$ *—is the spectral sensitivity of S receptors [-].*

To determine the resultant spectral sensitivity of each camera matrix, the summing process with weights was done using Formula (8).

(8) $$V_{C}\left(\lambda \right)=r\;V_{R}\left(\lambda \right)+g\;V_{G}\left(\lambda \right)+b\;V_{B}\left(\lambda \right)\;\left[-\right]$$

*where:*

$V_{C}\left(\lambda \right)$ *—is the calculated spectral sensitivity of the camera,*

$V_{R}\left(\lambda \right)$ *—* *is the spectral sensitivity of R-type pixel,*

$V_{G}\left(\lambda \right)$ *—* *is the spectral sensitivity of G-type pixel,*

$V_{B}\left(\lambda \right)$ *—* *is the spectral sensitivity of B-type pixel,*

r,g,b *—are fitting coefficients.*

In Formula (8), the fitting coefficients r, g, and b were individually selected to minimize the overall error f_1_′ determined from Formula (2). The selection involved using the trial-and-error method so that the error f_1_′ for the coefficients r, g, and b obtained the minimum value. The values of fitting coefficients are presented in Table 2.

**Figure 6:**
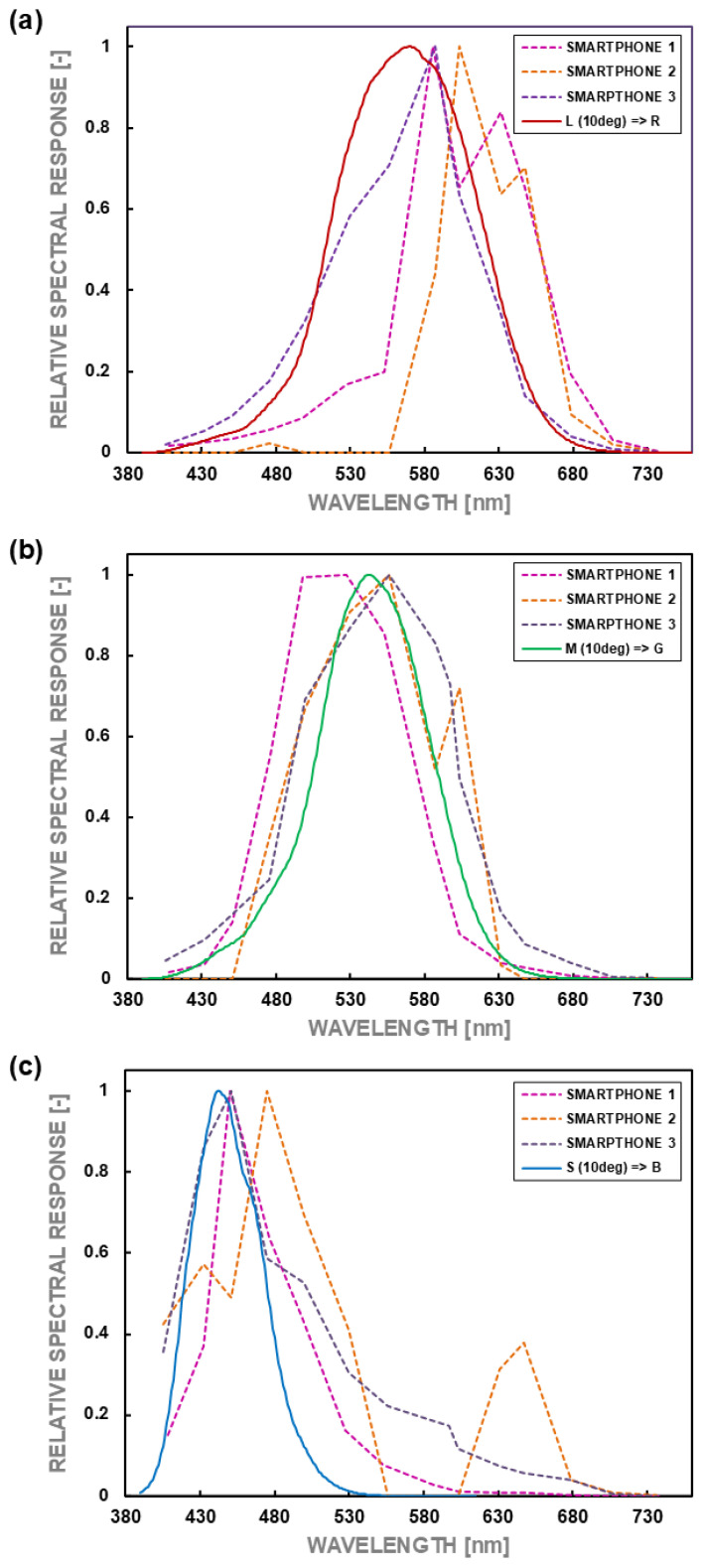
Matching measured R, G, and B spectral responses of particular smartphones to L, M, and S human eye receptor sensitivities: (**a**) to receptor L, (**b**) to receptor M, (**c**) to receptor S.

**Figure 7:**
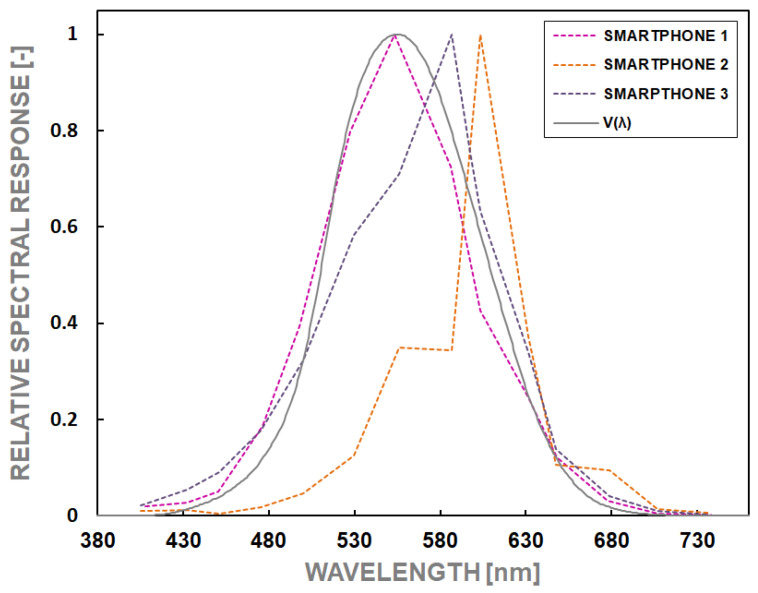
Matching calculated smartphone spectral responses to the photopic luminous efficiency function of a human eye.

The spectral power distributions of the individual channel sensitivity in the cameras tested are presented in Figure 6, and the summary of the determined characteristics V(λ) in Figure 7. The calculated values of the errors are presented in Table 2. Two values of the error f_1_′ for each camera are shown. They were calculated using a different reference. The first value, f_1_′(1), is the error calculated regarding the human eye’s theoretical photopic spectral efficiency function. The second value, f_1_′(2), refers to the eye sensitivity obtained from reference measurements made with a spectroradiometer.

The analysis of the spectral sensitivity measurement results showed that the errors in matching the spectral response of the individual channels are substantial and exceed 30% for each tested device. The values most similar to each other in individual channels occurred for Smartphones 1 and 3 (Table 2). On the other hand, for Smartphone 2, the smallest error (32%) occurred for channel G and the most significant error for channel B, amounting to over 130%. Considering such significant errors, it should be stated that each analyzed device should have an additional dedicated spectral correction (adequate filter) in order to increase the accuracy of luminance measurement. However, creating such a spectral correction filter is not a simple task and can only be implemented for the actual smartphone to be used. The spectral matching error f_1_′(1) for the tested devices is the largest in the case of Smartphone 1, about 98% compared with about 30% for the other devices. Smartphone 1 f_1_′(2) error decreases to just 16%, still compared to about 30% for the other devices. The magnitude of these errors is so poor in terms of spectral matching that it would not be appropriate even to classify these devices as low-quality photometers (Table 3).

The measurement method error *δ*M can be calculated by comparing the theoretical and reference eye sensitivity measurements using Formula (2) and substituting the appropriate values. It equals, respectively, 42.0%, 68.3%, and 41.1% for the measurement of Smartphones 1, 2, and 3. This demonstrates that the measurements are compromised by considerable uncertainty, exceeding the differences between the expected and realized spectral sensitivity curves of the matrix. Theoretically, the case of ideal overlap of the predicted and measured distribution is within the tolerance range, but a visual comparison of the individual sensitivity curve shapes shows that it is practically unattainable. It means that spectral mismatch errors exclude the tested devices to be used as universal luminance meters.

The research method should be changed to obtain better, more accurate results of the spectral sensitivity measurements of the matrices. More sophisticated laboratory equipment should be used. The separation of individual spectral bands with interference filters should be replaced by the dispersion of light in a monochromator. Similarly, the ordinary tungsten lamp should be replaced with a standard radiant energy source. However, this requires access to a better-equipped laboratory and causes the general costs to increase. Therefore, dedicated applications combined with individual calibration of cameras may give different results when used for other smartphones. In specific cases, reliable measurement results can be obtained when the use of a camera is limited to measuring radiation with a strictly defined radiation distribution, such as radiation emitted by electronic displays, but only after applying individual, complicated, and laborious calibration.

## 4. Conclusions

In this paper, the three high-end cameras installed in premium smartphones were tested. The measurement results show a clear difference in the data obtained, even in the case of smartphones from the same manufacturer but from different generations. Interestingly, a new generation of smartphones does not mean their use will necessarily be much better in photometric and mainly luminance measurements.

Our analysis of the linearity response measurement results of individual smartphones does not raise any major concerns. Smartphone 1 can be considered sufficiently linear, Smartphone 3 showed good linearity, while Smartphone 2 gives completely unpredictable results and is not suitable for performing luminance measurements. However, in the case of spectral matching analysis, none of the tested devices achieved satisfactory results in terms of measurement accuracy resulting from this parameter.

Our research also showed that universal applications must meet the expectations placed on them, even when only approximate values are required. Additionally, significantly different responses of different matrices can be observed at the same level of exposure. The matrix in Smartphone 1 is exposed 1.14 times more intensely than in Smartphone 2, and the response is 1.65 times weaker, i.e., the matrix is 2.23 times less sensitive. The case of Smartphone 2 showed that in some cases, even a dedicated application with the camera calibration option may not provide a reliable measurement result. Smartphone 3 can serve as a luminance meter in specific cases, provided a dedicated application is available, and the calibration is appropriately carried out.

The small size and negligible weight of digital cameras used in smartphones mean that they will be used in many measurement applications despite their clearly identified inaccuracy as described in this paper. The small aperture of the optical systems of these cameras and the very high resolution of the matrices, given their affordable price, will motivate further attempts to use them for measurement purposes, especially in the lighting area. The research initiated in this paper will be continued, to a limited extent, by using the matrix and optical system from Smartphone camera 3 to build a measuring station for testing imaging devices, primarily virtual reality equipment.

## Figures and Tables

**Table 1:** The comparison of basic technical parameters of selected smartphones.

PARAMETER	SMARTPHONE 1	SMARTPHONE 2	SMARTPHONE 3
**OPTICS**	own	own	Summilux
**SENSOR**	ISOCELL S5K2L4SX	ISOCELL HP2 200MP	LIGHT FUSION 900
**DYNAMICS**	10-bit	8-bit	12-bit
**BUYER FILTER**	No	Yes	No
**RESOLUTION.jpeg**	12.1 Mpx	200 Mpx	50 Mpx
**RESOLUTION.dng**	12 Mpx	12 Mpx	12.5 Mpx
**AVG PRICE (2024)**	~600 USD	~1100 USD	~1200 USD

**Table 2:** The values of fitting coefficients r, g, and b for each smartphone.

SMARTPHONE	r	g	b
1	1.13	1.00	0.00
2	1.21	1.00	0.02
3	0.79	1.00	0.01

**Table 3:** The obtained spectral mismatch errors of the tested smartphone matrix response.

**SMARTPHONE**	***f_1R_’*** ***[%]***	***f_1G_’*** ***[%]***	***f_1B_’*** ***[%]***	**PHOTOMETER CLASS**		
				*A: f_1_′ [%] < 3%*		
				*B: f_1_′ [%] < 6%*		
				*C: f_1_′ [%] < 9%*		
				***f_1_′ (1) [%]*** *	***f_1_′(2) [%]*** **	***Outcome***
**1**	77.2	51.0	85.7	97.8	16.6	Beyond the scale
**2**	80.7	32.1	131.2	32.8	34.6	Beyond the scale
**3**	62.2	48.4	48.8	38.2	32.7	Beyond the scale

* The reference is the theoretical spectral efficiency for the photopic vision, ** the reference is the spectral sensitivity based on measurements conducted with a spectroradiometer.

## Data Availability

Data underlying the results presented in this paper are not publicly available at this time, but may be obtained from the authors upon reasonable request.
